# The Cost of Safety: Balancing Biosafety and Histologic Integrity in Prion Research

**Published:** 2026

**Authors:** Alyssa Seerley, Andrea Grindeland Panter

**Affiliations:** 1 Weissman Hood Institute at Touro University; McLaughlin Research Institute, Great Falls, MT, United States

## Commentary

Prion diseases, or transmissible spongiform encephalopathies (TSEs), represent a group of incurable and invariably fatal neurodegenerative disorders driven by the pathological misfolding of the prion protein (PrP) [1-6]. These diseases are characterized by a progressive clinical course and, despite their significant impact on human [7-17] and animal [1,10,18-30] health, currently lack effective therapeutic interventions [31-34] and are limited in early-stage diagnostics [1,7,31-33]. Given the broad host range of prion diseases, together with the absence of effective treatments and limited antemortem diagnostic options, continued prion disease research remains essential. However, research and clinical investigations of prion diseases present substantial occupational biosafety risks [35,36] because infectious prion proteins are highly resistant to many conventional decontamination procedures. In fact, because prions are misfolded proteins rather than microorganisms, they exhibit exceptional resistance to conventional decontamination methods, exceeding that of most microbial pathogens [37,38]. As a result, specialized and rigorous prion inactivation protocols are required for minimizing potential exposure in laboratory and clinical settings [39,40]. Formic acid treatment has been demonstrated to reduce prion infectivity and is commonly incorporated into biosafety practices for the decontamination of prion-contaminated tissues [6,23,41] and materials, however, the morphologic effects of formic acid decontamination on tissues was not reported previously.

Our recently published study, Shabaan et al. [6], evaluated the morphologic effects of formic acid treatment on central nervous system (CNS) brain tissue using brain hemispheres from transgenic mouse models with variable degrees and patterns of neurodegeneration, see Table 1. The potential influence of age on tissue morphology following treatment was also assessed. Formic acid-treated tissues were compared with untreated control tissues in two experimental groups: young wild-type mice (4 months old; n = 3/group), aged wild-type mice (11 months old; n = 3/group), and 5xFAD mice exhibiting Alzheimer’s-like pathology (11 months old; n = 4/group). Tissues from mice with prion disease-associated spongiform degeneration were also evaluated; however, an untreated control group was not included because of biohazard considerations. In this cohort, Tg1536 mice expressing cervid *PRNP* at CWD endpoint (9.4 months old; n = 5) were compared with non-diseased control-inoculated mice that received normal brain homogenate (9.4 months old; n = 6).

Our study [6] analyzed CNS brain tissues treated with a standard formic acid prion decontamination protocol [41] to address the following:

1. Whether formic acid impacts tissue morphology and if so, whether tissue structure is maintained or too damaged for histologic studies.
2. Whether tissues with neurodegeneration and normal, non-diseased tissues are impacted similarly.
3. Whether morphologic changes induced by formic acid are distributed across brain regions.
4. Whether age of animals modulated formic acid impacts.

Corresponding brain sections (right formic acid, left control) were compared in the study, with one standardized section per condition used at the location of 1800 μm from the medial edge of the brain hemisphere. All samples were processed for histology simultaneously, with the right hemisphere subjected to the additional formic acid steps. The formic acid protocol consisted of a fixation step, a 95% formic acid immersion for 1 hour, a second fixation step as stated in Biosafety in Microbiological and Biomedical Laboratories (BMBL) [41] and then stored in PBS. For a detailed description of the formic acid and histologic methods stated, refer to the original manuscript [6]. Cortex width and hippocampal area measurements were performed with the experimental conditions unknown to the individual performing the analysis. Cortex thickness layers 1–6 were measured immediately dorsal to the CA1 of the hippocampus and hippocampal area measurements were performed by encircling the entire structure. Two separate measurements were performed in Fiji ImageJ^©^, were averaged for each structure as stated, and used for statistical analyses. The Student’s t-test determined statistical significance for pairwise comparisons unless otherwise noted in the original manuscript [6]. Results indicated that all brain hemispheres grossly appeared smaller with formic acid treatment with both the cortical width and hippocampal area measurements significantly reduced in the formic acid treated samples as compared to untreated controls. Additionally, tissue integrity appeared to be compromised as the appearance of tears and artifacts were observed more readily in the formic acid treated histologic specimens compared to non-formic acid treated samples. We determined that neurodegenerative pathology did not have a great effect on the amount of morphologic changes following formic acid treatment by comparing 5xFAD transgenic mice [42] with wildtype mice. These results demonstrated that similar reductions occurred in cortex width and hippocampal areas in both models after formic acid treatment. At the cellular level, formic acid treatment on the tissues did not appear to interfere with detection of neurons or astrocytes using immunofluorescence staining, however, the preservation of antigenicity, fluorescence intensity, or quantitative accuracy were not measured. When formic acid treated tissues exhibiting endpoint prion disease from Tg1536 [43,44] CWD infected samples were compared with uninfected Tg1536 tissues, the cortical width and hippocampal area measurements were almost identical, suggesting spongiform degeneration did not greatly alter or exacerbate cortical and hippocampal size reduction in the post-formic acid tissues. Formic acid treatment appeared to reduce tissue size differently based on brain region when cortex and hippocampus width ratios were compared in the combined wildtype and 5xFAD mice. Age did not appear to alter the effects of formic acid treatment on the samples, as there were no significant differences when comparing the cortex size reduction between the young and old mice. A summary of findings is provided in Figure 1.

The authors acknowledge that the limited number of mice included in this exploratory study may restrict the generalizability of the observed effects. Given the study design and sample size, biological variability among animals is difficult to assess, and the findings may not be universal. The results of Shabaan et al [6]. demonstrate that standard prion decontamination protocols commonly used in research and clinical settings can significantly alter CNS tissue morphology. Although the mechanism for this was not analyzed in the study, these may include lipid alteration or extraction, collagen alteration, acid-induced tissue contraction, and protein denaturation. Because formic acid is used in prion diseased tissues to substantially alter prion proteins and promote decontamination of the pathogenic protein conformation, protein denaturation is a plausible mechanism. Formic acid has also been shown to solubilize hydrophobic proteins found in the CNS, such as myelin [45]. Additionally, the study found that the reduction in size was not uniform across brain regions, with the hippocampus showing a greater decrease than the cortex. This may be due to variations in cell density or protein and lipid content in the specific regions.

Changes in tissue size and architecture, together with treatment-associated artifacts such as tearing, may confound histologic interpretation and reduce the utility of tissue specimens for diagnostic and research applications. Because reproducible and accurate histologic findings are essential for reliable interpretation in both clinical and research contexts, understanding the morphologic effects of prion decontamination procedures is important for improving diagnostic confidence and minimizing confounding artifacts. These findings also reinforce the importance of using validated prion-inactivation methods when handling potentially infectious tissues. In the absence of data defining the tissue effects of formic acid treatment, personnel may be tempted to avoid prion-inactivating procedures to preserve tissue morphology; however, the severe consequences of potential prion transmission underscore the need to prioritize biosafety while continuing to develop improved decontamination approaches that better preserve histologic quality.

Decontamination of prion-contaminated instruments, equipment, and surfaces using alternatives to formic acid have been studied [24,37-40,46-53], although, to our knowledge, no approaches have been developed specifically for histology applications. Standard decontamination techniques include extremely harsh techniques such as sodium hypochlorite (NaOCl) or sodium hydroxide (NaOH) treatments, sometimes in combination with 134° autoclaving under long periods of time [38-41,48-53]. Other methods investigated for prion decontamination include enzymatic solutions, alkaline cleaners, peracetic acid, phenolic disinfectants, and vaporized gases such as hydrogen peroxide [37,46,47,50,54], often with greatest efficacy when used in combination. Thus, there remains a need to develop additional methods that are compatible with histologic applications; however, identifying approaches that are both sufficiently gentle to preserve tissue morphology and effective against highly resistant prions remains a substantial challenge. Promising future research approaches to preserve tissue morphology while decontaminating prions may include replacing formic acid with novel, milder chemical treatments; incorporating tissue-protective additives; or modifying the existing protocol by evaluating the efficacy of reduced formic acid exposure times.

## Figures and Tables

**Figure 1.**
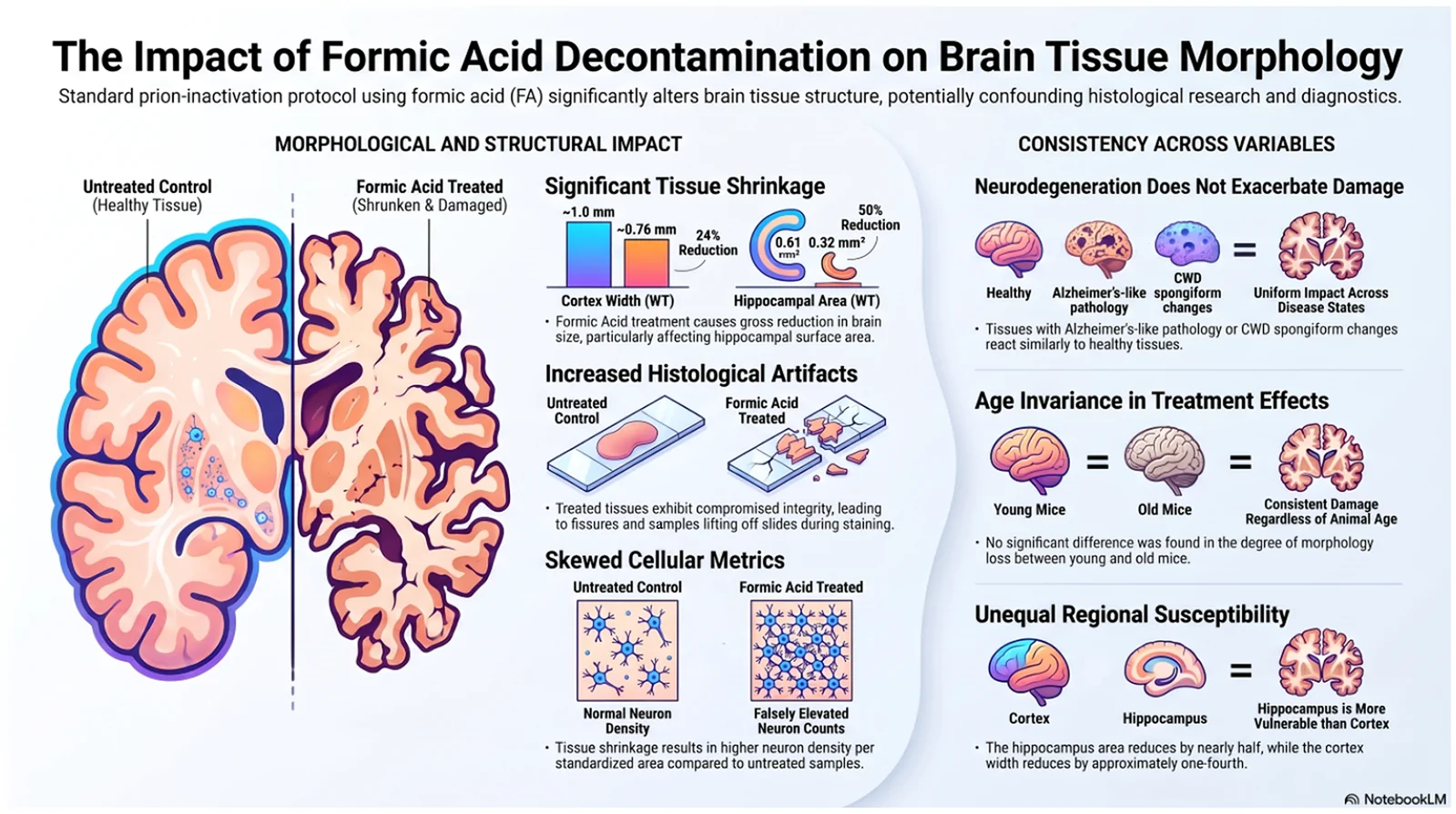
Overall results of study. Generated by AI platform, Notebook LM.

**Table 1.** Mouse models, conditions, and purpose for being included in the study of Shabaan et al. [6].

Mouse Model	Control Untreated		Formic Acid Protocol			Purpose
	Young (−4 months)	Old (~11 months)	Young (−4 months)	Old (~11 months)	Prion Endpoint (−9.4 months)	
WT C57BI/6N	3	3	3	3		1. Determine if formic (fa) treatment causes morphologic changes in brain tissues of wildtype mice 2. Determine if morphologic changes due to fa is altered depending on age of brain tissues in wildtype mice
5xFAD		4		4		Determine if fa morphologic changes are exacerbated in neurodegenerative disease mouse models when comparing fa vs untreated control
Tg1536 CWD					5	Determine if fa affects morphology differently between prion disease mouse brain tissue and non-diseased mouse tissues
Tg1536 NBH					6	
